# How Do Time Limits Affect Our Psychotherapies? A Literature Review

**DOI:** 10.5334/pb.475

**Published:** 2019-06-07

**Authors:** Rosa Maria De Geest, Reitske Meganck

**Affiliations:** 1 Ghent University, BE

**Keywords:** psychotherapy, time limit, termination, health care, ending

## Abstract

The application of time limits (i.e. restricting the amount of sessions before the beginning of psychotherapy) has become ingrained in psychotherapy research and in the context of managed care, mostly due to pragmatic and economic reasons. However, little is know on how this technique interferes with the psychotherapeutic process. Although several theorists have considered the possible advantages and drawbacks of the technique, research explaining these mechanisms is scattered. By conducting this review, we strived to answer two questions: (1) Does a time limit alter the psychotherapeutic process? And (2) In what way? In doing so, this study aspires to grant more insight into the mechanisms of time limited psychotherapy and aimes to contribute to a first understanding of the dynamics of a time restricted therapy process. We searched for articles in the databases of Web of Science and Pubmed. Our review identified 28 studies that provide empirical grounds to explain processes involved when applying a time limit to psychotherapy. Qualitative research suggests that a time limit exerts pressure on the therapy process and creates an expectancy effect, which can have both positive and negative consequences. Additionally, time limits can be associated to therapists taking on a more directive role in therapy. Results show that a time limit is anything but a neutral intervention; it is a technique that complexly interacts with therapy processes on multiple grounds. Further research is vital to determine which environment is appropriate for its application.

## Introduction

In our study, time limited psychotherapy (TLP) is defined as any type of psychotherapy that sets a time restriction at the beginning of psychotherapy. This review aspires to help establish an empirically grounded theory on TLP by collecting research results on the mechanisms involved in a time limited process. A time limit is currently used on a large scale in our society (for example in research and in managed care), although very little is known regarding the technique. Alarmingly, early studies have shown that applying a time limit can have negative consequences (Murphy, Debernardo, & Shoemaker, 1998). This gives us all the more reason to take a closer look at a technique that is too often considered as a neutral to the psychotherapeutic process.

### A brief history of time limited therapy

The use of a time limit dates back to the very beginning of psychotherapy as a formal practice, namely the first half of the 20th century. In his treatment of the ‘Wolf Man’, Freud (1918) decided to limit the length of the therapy, after having worked with the patient for an extensive period of time because he felt the progress had been hampered. The case illustrates how Freud was attempting to shorten therapies towards the end of his career. Freud’s early treatments were actually brief and symptom-focused, however, this changed when he adjusted his initially active technique to a more neutral stance and let his patients engage in free association. As a result, therapies were growing longer and longer, and consequently became a long-term luxury for the privileged (Groves, 1996; Hoyt, 2005).

Psychoanalysts such as Sàndor Ferenczi and Otto Rank also attempted to counter this trend of exceedingly long therapies by experimenting with more active techniques (Hoyt, 2005; Safran, 2002a). In the wake of this evolution, several new and more active ‘brief’ therapies saw the light of day, including some with a time limited feature. ‘Brief therapy’ and ‘time-limited therapy’ are often mentioned in the same breath, and are inevitably intertwined as is seen in the history of TLP. They are, however, not equivalent to one another. Various practitioners and theorists who work with time limits in their practices have suggested that TLP may encompass a very different psychological process than open ended therapy no matter how brief (Johnson & Gelso, 1980). Overall, the terms ‘brief’ or ‘short-term’ therapy also imply more than the application of a time limit. Brief therapy is known for its focus on efficiency by applying several distinct techniques such as a ‘here and now’ principle, a decisive focus in therapy and the emphasis on patients’ strengths. A time limit is not per se included, but fits within the ideology of brief therapy.

As mentioned above, time limited psychotherapy is defined purely technical in our study as any type of psychotherapy that sets a time restriction at the beginning of psychotherapy. We therefore included all types of psychotherapy that exert a time limit in our review, whether they were labeled “brief” or not. This way, this study focuses goes beyond therapeutic orientations to study the technique itself.

Several brief therapy pioneers, such as James Mann (1973), David Malan (1976) and Peter Sifneos (1979), have integrated a time limit in their short therapies and reflected on the effect it had on the process and their patients. For example, James Mann (1973) hypothesized that the intervention provides structure and motivation for patients and can accelerate the therapeutic process. Unfortunately, these theoretical hypotheses have been hardly examined through empirical research. Since the 1980’s, the focus in research shifted more and more towards proving the efficacy of short-term treatments, while questions on the working mechanisms of a time limit in psychotherapy were relinquished to the background.

### The ideal dosage of therapy

Since the 1980’s the quest to find an optimal dosage of psychotherapy became an increasingly popular research topic. In an article named ‘The Dose-Effect Relationship in Psychotherapy’, a group of researchers (Howard, Kopta, Krause, & Orlinsky, 1986) systematically attempted to specify the number of therapy sessions required for the majority of patients to show improvement. According to their results, by eight sessions approximately 50% of patients were measurably improved, and approximately 75% were improved by 26 sessions. The researchers indicated that these estimates could be seen as a guideline for applying time limits in practice, although they did not represent maximum therapy benefits (Howard et al., 1986).

In the years to follow, these dose-effect numbers were criticized in several ways. First off, Hansen, Lambert, & Forman (2002) argued that the estimations of the Dose-Effect study were not based on data from naturalistic settings and found that when the research was conducted in a naturalistic setting, only 20% of patients improved with an average number of five sessions. According to their review, treatment limits should be set well beyond 20 sessions if more than 50% of patients are to experience a clinically significant gain. Additionally, Feaster, Newman, and Rice (2003) demonstrated that the traditional research designs of most dose-response studies contained important methodological flaws. According to the authors, many of these approaches have conceptually confused two distinct concepts, namely, do participants with different characteristics need different amounts of therapy and do otherwise similar participants show different outcomes when given different levels of a particular type of therapy? Because of these methodological shortcomings, they state that current research findings are not sufficiently pervasive to provide a sound scientific basis for setting policies about the implementation of a time limit in mental health services. More recently, two studies (Baldwin, Berkeljon, Atkins, Joseph, & Nielsen, 2009; Stulz, Kopta, Lutz, Minami, & Saunders, 2013) found that patients’ rate of change varied in function of the total dose of treatment. The results showed that small doses of treatment were related to relatively fast rates of change, whereas large doses of treatment were related to lower rates of change. The researchers advocate that in naturalistic data, dose should not be seen as a predictor of treatment response but rather as a marker of the speed of treatment response of the patient. Given the variability in rates of change of patients, the researchers conversely stress that uniform time limits for treatment for all patients would not adequately serve individual patients’ needs. Unfortunately, it remains unclear whether this variability in treatment responses can be explained by differences in patients’ characteristics. Research suggests that higher distress levels at intake, higher baseline distress and the presence of characterological symptoms could account for a slower rate of change. Baldwin et al. (2009) hypothesize that the variability in rates of change may be explained by differences in patient variables, therapist variables, treatment variables, or a combination of the three.

To conclude, the past decades have seen a significant amount of research conducted to justify an efficient yet adequate number of therapy sessions to apply to the larger public. However, a simple answer to this question is yet to be found. As Shapiro et al. (2003) stated in their brief therapy research review: ‘Current research does not begin […] to warrant any rigid prescription of a universal time limit for psychological treatment’ (p. 231). Strikingly, time limits are nevertheless frequently applied in current mental health practices, as we will discuss in our next paragraph.

### Current use of time limits in research and managed care

Within the context of psychotherapy research, fixed treatment durations are regularly applied. Time limits are set in the majority of randomized controlled trials (RCT) to minimize within-group variability, but also because of cost containment and feasibility issues. In this setting, the time restrictions are purely set out of pragmatic reasons external to the therapeutic process, and have little to do with altering or ameliorating this process (which was for example the case in the therapy of Freud’s ‘Wolf Man’, as mentioned above). In research conditions, pragmatic reasons for applying a time limit undeniably prevail, and are focused on increasing internal validity through standardization. However, it has often been criticized that this quest for internal validity comes at the cost of external validity (Blais & Hilsenroth, 2007). According to Blais and Hilsenroth (2007), time limits can be seen as part of this validity problem, since a fixed treatment duration does not match a substantial part of clinical reality. Researchers stress that in everyday practice, actual treatment duration is highly variable compared to the 11 to 20 sessions set in RCT trials. Although the critique on the RCT format seems to have had its effect on the psychotherapy research field (as we can see for example in the growing interest in qualitative research), the use of time limits in psychotherapy research is hardly questioned. Most researchers continue to regard a time limit as a neutral intervention that does not affect their studies or therapies. The results of our review will show that this is most likely an untenable idea, since a time limit may affect the therapeutic process on multiple levels. Unfortunately, very little research has been conducted on the working mechanisms of TLP. As we mentioned above, since the 1980’s, research focus has shifted away from the specific mechanisms of time limited treatments towards outcome-related aspects of treatment delivery (Shapiro et al., 2003). As a result of the emphasis placed on providing an empirical ground for the effectiveness of TLP, we currently know that it can be effective (Abbass et al., 2014; Johnson & Gelso, 1980), but not what the effect of a time limit on the therapeutic process is.

This lack of knowledge is increasingly worrying since the use of time limits is becoming more and more the standard within the context of managed care. Health care systems worldwide are under increasing pressure to optimize their cost-effectiveness (Shapiro et al., 2003) and TLP contains the promise to achieve results with a smaller amount of time and expense. Certain aspects of the controlled research setting, such as a time limit and manualization, have therefore found their way into everyday practice. However, this is not because time limits have been investigated extensively or have showed to improve clinical practice, but mostly out of pragmatic considerations. And while on the one hand, studies show that TLP is an effective treatment (Abbass et al., 2014; Johnson & Gelso, 1980), on the other hand, certain studies in managed care settings have shown alarming results, namely that restricting the amount of therapy sessions can have negative consequences and lead to inappropriate treatment, insufficient treatment, or both (Murphy, Debernardo, & Shoemaker, 1998). The research focus on the efficacy on TLP (through RCT’s) has given us very little insight into this phenomenon, because these broad group results tell us little about the dynamics of a time limited process.

By collecting qualitative and quantitative findings on the subject, our review aspires to provide a first insight into the effects and (dis)advantages of a time restricted therapy process. The purpose of the current study was to scrutinize existing literature with respect to two critical questions regarding the processes involved in TLP. Firstly, does a time limit alter the psychotherapeutic process, and secondly: in what way? Altering the psychotherapeutic process is defined here as influencing the therapist, patient and their interaction in psychotherapy and affecting the therapies’ change mechanisms.

This review provides a unique contribution to the existing research literature because a) it is the first thorough overview on TLP literature since the 1980’s (Johnson & Gelso, 1980) and b) it is the first one ever to focus specifically on the processes involved in TLP, rather than focus on outcome. Moreover, this review focuses on how a fixed ending alters psychotherapy, and not how differences in duration affect psychotherapy. Namely, our research question does not encompass whether short-term therapy is better than long-term therapy, but whether TLP entails a different process than open ended therapy, no matter how long or short.

## Methods

**Figure 1:**
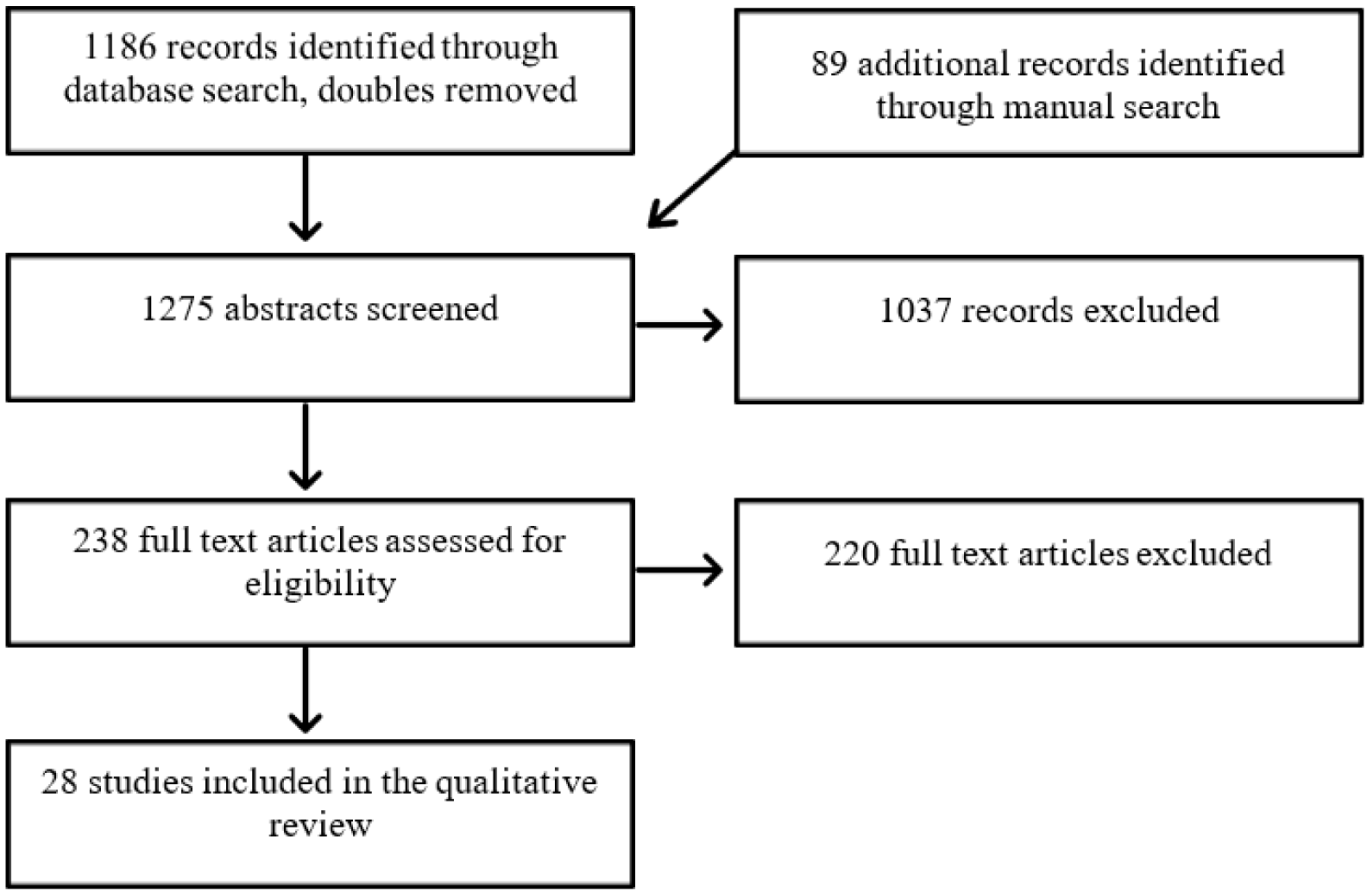
Flow chart of the selection process.

We conducted a search in the databases of Web of Science and Pubmed combining the terms ‘Psychotherapy’ AND ‘Time Limit’. This process was supplemented by a manual search of references cited in retrieved articles. Out of the 1275 results, we selected 28 articles (of which 17 came from our original search, and 11 from the manual search), using the following criteria: a) the abstract or title should contain a focus on the working mechanisms of TLP, b) the research focus was individual psychotherapy, excluding group therapy, c) we only included studies with an adult (18+) population, and d) articles should be in English. To the extent of providing a critical and comprehensive exploration and discussion of research findings, we included both empirical and theoretical studies. As RCT’s almost exclusively investigate time-limited therapies, we scrutinized all RCT’s we came across in our search, however the great majority of these studies paid no attention to any possible effects of the time limit on the therapeutic process. Time criteria included articles between 1955 and 2019. The peer-reviewed nature of journals listed in WoS and Pubmed served as a basic quality guarantee for our review. Furthermore, we appraised the methodologies of the retrieved articles by reviewing their reliability and validity. Figure 1. depicts a flow chart describing the selection process of the articles.

We used principles of thematic analysis (Braun & Clarke, 2006) to identify patterns within the data. Thematic analysis is a method for identifying, analysing, and reporting patterns within data. We applied this technique to find patterns in the articles that discuss the mechanisms involved in TLP. This was done by identifying and coding relevant features in the data, followed by searching for themes within these codes. We reviewed, refined these themes and returned to the initial codes to validate them (or adjust them if they were no longer in line with the initial codes). This process was supervised by the second author of this study.

## Results

We delineated five categories in which the mechanisms of time limited therapy could be arranged: **‘therapists alter their therapeutic approach’, ‘patients’ experiences, ‘acceleration of change’, ‘reducing dropout’** and **‘patients’ characteristics**, and organized the literature accordingly.

### Therapists alter their therapeutic approach

We have found several studies that cast a light on how therapists perceive working with a time limit in their daily practice. Early qualitative studies in the 1980’s have sketched a broad picture of their experiences. For example, Johnson and Gelso (1980) found that client ratings and different types of psychological tests favored TLP over time unlimited treatment, while the counselors themselves felt less inclined towards it. The researchers hypothesize that while to some extent the counselors’ more negative evaluation of TLP may demonstrate their prejudices towards time limited treatment, the various measures could also reflect different facets of improvement. More specifically, counselors may prefer open ended treatment because of its greater opportunities to work toward insight and personality reconstruction, while clients themselves may be content with TLP because it helps them feel better rapidly (Johnson & Gelso, 1980). In another survey, Burlingame & Behrman (1987) pointed out that TLP was perceived as more efficacious by therapists in cases of situational adjustment reactions, but the therapists favored unlimited treatment over TLP for several other diagnoses such as neurotic depression, psychosis and personality disorders. The researchers suggest that therapists often see TLP as a crisis intervention, therefore being more applicable to disorders such as a situational adjustment disorder.

While these early qualitative studies give us an idea of broad feelings therapists foster towards TLP, other studies describe therapists’ experiences more in depth and can be categorized according to two settings in which time limits are frequently used – research and managed care -we will start by discussing the latter type of studies.

#### TLP within the context of managed care

In the USA, TLP is often utilized within the context of managed care. In this case, managed care companies decide how many sessions will be reimbursed for a specific client (mostly based on diagnosis), as we discussed in the introduction. Remarkably, a widespread quantitative survey of clinical psychologists showed that 79% perceived that managed care had a negative effect on their professional work (Phelps, Eisman, & Kohout, 1998). In a more detailed questionnaire (Murphy, Debernardo, & Shoemaker, 1998), four out of five psychologists in independent practice indicated that putting limits on the of the number of sessions interfered with their treatment. The research showed that the caps on the sessions were one of two variables (the other one being the clinician’s loss of control over making treatment decisions, which can also be related to the decision over the length of therapy) that strongly contributed to concern about quality of care. These studies point out that a vast amount of therapists question the use of time limits in psychotherapy. Below, we will discuss two qualitative studies that discuss more extensively how this influence from managed care is perceived by therapists.

First off, Cohen, Marecek, and Gillham (2006) interviewed 18 therapists from different approaches working under managed care plans in the USA. More than two thirds of the therapists reported that the lack of control over the length of treatment^1^ was an obstacle to its successfulness and they often felt therapy ended before the client was ready. The time constraints also made them alter their therapeutic approach: they were compelled to focus on superficialities without addressing underlying problems. This shallow approach was a source of frustration and also raised ethical concerns for the therapists, who felt obligated to provide treatment they regarded to be inadequate. A more recent study conducted in Australia showed similar results (Wright, Simpson-Young, & Lennings, 2012). The researchers sent out questionnaires and interviewed Australian psychologists^2^ working with a time limit that ranged from one to seven sessions. These therapists also experienced the structure and planning of TLP differently from therapy without a time limit. Overall, there were two broad differences in their approach – participants said that, without limits on the number of sessions, they would ‘do more’; they would address more content, go into the issues of the patient more in-depth, try to address additional historical-contextual factors, do more thorough assessment and combine different approaches. Secondly, they would also ‘work differently’ – they would be less symptom-focused, less problem-focused, more client-centered, and the need to refer the patient on would be less frequent (Wright et al., 2012).

It is striking how in both studies (Wright et al., 2012; Cohen et al., 2006), that were conducted under different circumstances, therapists indicated that their therapies differ when working with a time limit under managed care. Therapists in both contexts struggled with the fact that their therapies became more superficial because of the time limitations. On a broader level, Cohen et al. (2006) state that the results highlight how therapists and managed care companies have contrasting interests: while the therapists take a long-term perspective on therapy in which the prevention of future difficulties is crucial, the spirit of managed care seems to prefer a short-term view in which the goals were based on superficial behavioral criteria and symptom remission. The therapists’ holistic view on a person that accounts for a complex combination of elements such as personal character, moral personhood, social relations, and personal growth is sidelined in the context of managed care. In their study, more than half of the therapists also raised concerns that communications from managed care personnel led clients to an image of therapy that was detrimental to treatment (Cohen, Marecek, & Gillham, 2006). Therapy was described by managed care personnel as if it were ‘a time-limited, one-size-fits-all procedure akin to a dental checkup’ (p. 256). Clients often assumed that the number of sessions authorized was the number sufficient for a cure. Clearly, such a view is not without consequence on the patient’s engagement in therapy and the therapeutic process. More specifically, the therapists felt that practices like these deny the need for clients to take an active part in the therapy (Cohen et al., 2006). In the study of Wright et al. (2012), a similar concern was articulated: when using a time limit, therapists saw increased directiveness as necessary to achieve the pace required to meet goals within the expected time frame. An effect of such directiveness was that, for some participants, there was a concern that clients did not have sufficient control over the therapeutic process. In both studies the therapists are thus troubled that a time limit impedes clients from being in charge of their own therapy process.

In addition to these concerns, some therapists in the study of Wright et al. (2012) felt that the therapeutic alliance was threatened by the imposed time limits. Others felt, however, that it was possible, in TLP, to speed up the formation of the therapeutic alliance.

#### TLP within a research setting

Since it is considerably more difficult to do standardized research on long-term psychotherapy, psychotherapy researchers tend to prefer the time-limited psychotherapy format because of cost containment and pragmatic reasons such as the aforementioned standardization of the research context. These considerations have had a tremendous impact on the entire field of psychotherapy research, resulting in an abundance of outcome studies on time limited therapies and a lack of meaningful research on long-term psychotherapy (Safran, 2002b). In this paragraph, we will focus on the experiences of therapists who cooperate in this psychotherapy research.

Busch et al. (2001) interviewed therapists working within a time frame in a research setting. The therapists participated in an RCT on the effect of psychodynamic treatment on panic disorder. Here, the time limit was set much broader (24 sessions) than in the study of Wright et al. (2012), where the time limit ranged from one to seven sessions. Nonetheless, the therapists expressed concern from the beginning of the study that the time length of the therapy was not in accordance to the psychopathology the patients presented. In this specific study, the therapists were concerned that the time limited aspect of the treatment would contribute to the recurrence of panic attacks after the treatment since they were not given the time to address the full dimension of their patients neurosis.

Surprisingly, the pressure that was created by the time constraint had its pro’s and cons. On the one hand, it fostered feelings of inadequacy in the therapists and as the termination drew near, this often created an upsurge of tension in both patients and therapists. On the other hand, because of the time limit, the patients separation issues were addressed early on in therapy and this allowed for a productive focus on these issues as termination approached. Reassured by the positive results of the study (a remission rate of 97%), the therapists felt more confident and less conflicted towards participating in psychotherapy research. The authors suggest that psychoanalysts might have been overestimating the potential damage of research constraints on psychoanalytic process and outcome (Busch, et al., 2001). Unfortunately, we found no other qualitative studies that replicate or elaborate on these findings.

To summarize, this theme focused on how therapists perceive the impact of a time limit on the psychotherapeutic process. In both the research and managed care setting, therapeutic approach was altered because of the time limit. More extensive research is paramount in these contexts to draw decisive conclusions on the subject. Future research should bring into account the number of sessions set for a specific setting: in the studies we discussed, the time limit could vary from one to 24 sessions. The different lengths of time limits might affect psychotherapists, patients and their therapies differently.

### Patients’ perspective

While psychotherapy research only marginally explored the therapists’ perspective, studies on how patients experience a time limit in psychotherapy are even scarcer. In their review on the effectiveness of TLP in 1980, Johnson & Gelso combined the results of several studies exploring the patients’ perspective on TLP and found that their experiences with TLP were generally more positive than those of the therapists. Although TLP clients seemed to be satisfied with their counseling in general, multiple studies found them to be less satisfied with the length of treatment both after termination and at 18-month follow-up (Johnson & Gelso, 1980).

These findings are supported by Dekker et al. (2005) in an RCT in which patients were questioned on how satisfied they were with the number of psychotherapy sessions. Clients had received 8 or 16 sessions of PDT or CBT. Remarkably, three quarters^3^ of the therapists considered the number of sessions to be sufficient, while only half of the patients shared the same opinion.^4^ In total, approximately 43% of patients thought there were not enough therapy sessions. This applied equally to both treatment conditions.

Having found only two studies considering patients’ experiences with a time limit, we find it troublesome that patients, who are the central agent in psychotherapy, received so little say-so on the subject. Although we cannot make any definite conclusions based on two studies, the results seem to emphasize that a large group of patients might feel uncomfortable with a time limit in psychotherapy.

### Acceleration of change

According to several authors, the application of a time limit is one of the features of short-term therapy that enables an acceleration of therapeutic change (Eckert, 1993; Johnson & Gelso, 1980; Messer, 2001). Clients and therapists are thought to be encouraged to work harder and faster with the time limit in sight. Messer (2001), for example, specifies that a deliberate time limit can add a sense of intensity and urgency in therapy and activate therapist and patient expectancies as to when change will occur. On behalf of the therapists, the time limit would require them to be more active in therapy and to maintain a specific focus, leading to a quicker result. On behalf of the patients, some refer to Parkinson’s law, which states that a task expands itself so as to fill the time available for its completion. Translated to psychotherapy, patients are said to ‘shrink the time necessary to perform a task when little time is available, or expand the time work takes when more time is available’ (Rasmussen & Messer, 1986: 427). In the following paragraph we will look at research that supports the idea that the application of a time limit can accelerate the therapeutic process.

In 2003, Shapiro and his colleagues made a comparison between 8 versus 16 sessions of TLP for two different types of therapy: cognitive behavioral therapy and psychodynamic interpersonal therapy. The study was called the ‘Second Sheffield Psychotherapy Project’ (SPP2) and its results showed that by the time all clients (diagnosed with depression) had received eight sessions of treatment, improvement^5^ in terms of depression symptoms was significantly lower for the 16-session clients than for the 8-session clients (who had by then concluded their therapy). The results therefore suggest an acceleration of symptom reduction in the 8-session condition.

The results of the SPP2 study are backed up by a comparable study in the Netherlands (Dekker et al., 2005). Here, a similar comparison was made between 16 and 8-sessions of short psychodynamic therapy. The results showed only small differences between the conditions at the various measurement points.^6^ These little variations also indicated that remission levels were higher around week 8 of the 8-session condition compared to the 16-session condition.^7^ After 6 months, no more differences between the 8 and 16-session conditions were found. Dekker et al. therefore draw the same conclusions as in the SPP2 study: they find it conceivable that patients in the 8-session condition adopted a more active attitude because they only had 8 sessions in which to bring about an improvement (Dekker et al., 2005).

While the outcome results on the Beck Depression Inventory (BDI, Beck, Ward, Mendelson, Mock, & Erbaugh, 1961) seemed to change more quickly in the eight-session condition and showed the same results at the finish for both conditions, Dekker et al. (2005) found a different evolution on the Inventory of Interpersonal Problems (IIP, Horowitz, Rosenberg, Baer, Ureno, & Villasenor, 1988). On this questionnaire, virtually identical proportions had improved in the two treatment groups by the eight session. Yet while 40% of clients had improved at the end of the 16-session treatment, only 18% showed improvement in the eight-session treatment. Thus, on an interpersonal level, an acceleration in improvement could not be found. While the length of a time limit seems to have an effect on the speed of change in symptom improvement, more thorough changes on an interpersonal level appear to be harder to influence through this variation.

On a different stance, several authors (Hill, CHui, & Baumann, 2013; McLeod, 2013b) have challenged the notion that changes in scores across psychotherapy accurately reflect changes as a result of therapy. According to Mcleod (2013b) we should also take into account the fact that clients complete questionnaires differently depending on their context. This phenomenon is called the ‘hello goodbye effect’, which describes how, at the beginning of therapy, patients’ responses to questionnaires reflect their desperation and need for help. Mcleod proposes that the therapy process itself can alter how clients perceive these questions and can cause them to respond differently at the end of therapy, even though they may feel the same as before. We can therefore question whether the variation in time limits has an actual effect on the speed of the therapeutic process, or whether the results are biased by a weakness in the validity of the questionnaires.

From the therapist’s point of view, coherent patterns were found in the changes in therapeutic focus across sessions. More specifically, on the dimension of how therapists worked towards client change, the SPP2 study showed that when the psychodynamic therapists only had eight sessions in therapy, the last four sessions revealed a steady rising emphasis on encouraging change. In contrast, the 16-session psychodynamic therapies were characterized by a gradual increase over the whole course of therapy until the final two sessions prompted therapists to encourage change just as much as in the closing stages of eight-sessions psychodynamic therapy. The therapists in the eight-session condition thus seemed inclined to start focusing on change more quickly in therapy. This is in line with the theoretical assumption that therapists are required to be more active in a more time limited therapy.

In both the SPP2 study and the study of Dekker et al. (2005), a comparison was made between two time limited conditions. Although this does give us an idea of the different effects of different lengths of time limits, it does not enable us to draw conclusions on the difference between time limited and open ended therapies.

Some authors suggest that pressing therapists to lay a rigid focus in therapy can have its downsides. According to Hatcher, Huebner, and Zakin (1986) a danger in TLP is that the therapist’s activity combined with a rigid adherence to a specific focus might mask the fact that an inappropriate focus has been chosen. The results of their study showed that over the course of brief therapy, the proposed focus was shaped, refined and at times even entirely changed. The researchers therefore argue for flexibility in the management of a short-term focal treatment. Although these results should be replicated with a larger population to be more conclusive, the authors nonetheless introduce a challenging idea: when therapists in TLP adhere an overly rigid focus, they are in danger of losing connections with other crucial themes in the client’s narrative. We can relate this hypothesis to the experiences therapists had while working with time limits in managed care: they felt they had to focus on superficialities without being able to address underlying problems. We therefore wonder which conditions are ideal for therapists to feel comfortable setting a focus and working towards it in a specified number of sessions. In a worst case scenario, it seems as if this focus can shift towards a situation in which therapists become anxious and feel as if they do not have the time to address the origin of the patients problems in therapy. On a different stance, Migone (2014) questions the rationale behind a time limit. If time limits exist to make the patients work harder and faster, he says, we should first of all try to understand why the patients should be pushed to do so, and not simply bypass their resistance with a time limit.

### Dropout

Dropout rates in psychotherapy are commonly reported to be strikingly high. An important meta-analytic review from Wierzbicki & Pekarik (1993) reported an average dropout rate of 47% across 125 studies. The majority of patients that drop out of therapy do so in the first sessions. The phenomenon is generally perceived as problematic, especially within the healthcare system, where resources are mostly scarce.

Interestingly, the numbers on dropout vary widely across studies and settings. The interval is assumed to lie between 30 and 50% (Roos & Werbart, 2013). Some researchers explain a part of this variety by suggesting that time limited therapies have lower dropout rates than open-ended therapies (Deykin, Weissman, Tanner, & Prusoff, 1975; Messer, 2001; Ogrodniczuk, Joyce, & Piper, 2005; Pekarik, 1985; Reder & Tyson, 1980). They have several hypotheses on how a time limit could influence the psychotherapeutic process. Firstly, Sledge, Moras, Hartley, & Levine (1990) argue that patients would be more inclined to remain in therapy because a time limit provides a psychological structure, helping patients face the difficult feelings and experiences psychotherapy can provoke. They suggest that when the end is in sight, patients may be more willing to ‘face the music’ because of the time limit. Pekarik (1985) on the other hand proposes that clients might expect and desire a shorter treatment duration compared to what their therapists foresee for them. According to him, the lower-dropout rate in TLP could be attributed to a congruence between the patients’ expectations and the time limited therapy format.

Other, alternative explanations attribute the lower dropout rate in TLP to other variables than the setting of a time limit. For example, Ogrodniczuk et al. (2005) argue that the lower dropout rates for short-term therapies could also be function of time itself: patients in short-term therapy simply have less occasion to terminate prematurely compared to patients in long-term therapy. Messer (2001) suggests that certain aspects of short-term therapy such as the therapist’s working to maintain a focus, establishing a collaborative working relationship, and being quite active can be a factor that contributes to a patient’s decision to continue therapy, aside from the setting of a time limit.

In the past, several studies that compared dropout rates for open-ended and time limited therapies were often statistically and methodologically flawed (Swift & Greenberg, 2012). Often other variables besides the time limit were not held constant, such as the specific form of therapy or the characteristics of the patients participating (Sledge, Moras, Hartley, & Levine, 1990; Straker, 1968; Deykin, 1973). Because of these methodological shortcomings, it is uncertain if the differences in drop-out rates in these studies can be attributed to the time limit itself. A recent, rigorous meta-analysis on the causes of dropout rates (Swift & Greenberg, 2012) shows that significantly higher dropout rates can be found for open-ended than time-limited treatments (29% versus 17,8%). However, in their discussion, the researchers propose that this result may be caused by the controlled conditions of efficacy studies: for example, patient groups are often selected for their suitability wherein, for instance, patients with a personality disorder are left out. If patients who are more probable to have a difficult trajectory are left out, it is no surprise that these studies show lower dropout numbers. Yet these studies are often the ones that also have a time-limited feature. We therefore do not know if the difference in dropout rates between open-ended and time-limited treatments is due to the intervention of a time limit, or to other characteristics of a randomized controlled trial (Swift & Greenberg, 2012).

### Client characteristics

In his handbook for short-term dynamic psychotherapy, James Mann (1991) articulates that the contraindications for TLP are quite clear: certain diagnostic categories – such as severe forms of depression, schizophrenia, bipolar disorder, borderline personality and schizoid characters – a priori demand indefinite long-term involvement with the patient and therefore do not correspond well with a time limited format. He also highlights the importance of the patient’s ego strength as measured by previous work performance and past relationships. This excludes patients who might have difficulties engaging and disengaging rapidly from treatment, such as schizoid patients, certain obsessional patients, patients with strong dependency needs and some narcissistic patients (Ursano & Hales, 1986). In their review in 1980, Johnson and Gelso found evidence that supports the assumption that less well-adjusted, more chronic clients may do better in open-ended psychotherapy than in TLP. A study by Thase et al. (1992) also supports this hypothesis. Here, 48 patients diagnosed with depression were examined during and after a time-limited course of cognitive behavioralt therapy. Results showed that a large group of patients (32%) relapsed during a one year follow-up. In our interest, a number of variables that correlated with relapse were identified: these patients tended to be suffering from a recurrent episode of depression and reported higher levels of depressive symptoms and dysfunctional attitudes when they entered the study. Besides these characteristics, they also ended the time limited treatment with higher levels of residual symptoms and showed a slower response during therapy.

A different type of study of Vinnars et al. (2007) examined whether certain traits of personality disorders (PD) predicted treatment outcome in time limited and open-ended treatment. The results showed that with higher levels of dominance, patients being treated with non-manualized therapy (i.e. therapy that does not use of any form of standard manual) had the highest rate of change. The researchers suggest that the active stance of therapists in time-limited, manualized psychotherapy is not complementary with patients with highly dominant personality traits.

Regarding qualitative research on the subject, two case studies describe the process of TLP with a patient with a ‘more severe’ diagnosis that would usually counterindicate a time limited therapy, namely schizophrenia (Thomas, 2017) and narcissistic issues (Binder, 1979). In the case study of Thomas (2017), the author describes how the intense movements of closeness and separation (that are inherent to TLP) were more difficult for her patient, who had paranoid tendencies. However, the author hypothesizes that she was able to override the patients’ paranoia by taking interest in the person behind his diagnosis and actively seeking out the healthy, human aspects of her patient. The case study of Binder (1979) describes a less positive course of TLP with a client having narcissistic issues. According to psychoanalytic theories, patients manifesting significant narcissistic difficulties exhibit such stubborn character defenses that they are very hard to alter. The case study appeared to confirm this hypothesis. According to the author, his patient (unconsciously) experienced the time limit as irrevocable loss and abandonment, and dealt with this inevitability by devaluing him as a therapist. In the end, the author suggested to refer the patient on towards long-term psychotherapy.

While these qualitative case studies provide some insight into the interaction of TLP features with patient characteristics, we are hesitant to draw conclusions, as their methodology lacked transparency. More extensive research on the subject is paramount to be able to understand which clients are suitable for TLP and which are not, as it is clear that client characteristics interact with all dimensions of therapy and also with an important intervention such as setting a time limit. Future studies could also explore how patients with severe separation issues might be affected by a therapy in which the end is definite and irreversible.

## Discussion

### Summary

While scanning the literature on the possible effects of a time limit on the psychotherapeutic process, it became clear that there is a vast lack of research material on the subject. Therefore, we are limited in the conclusions we can draw based on our results. However, returning to our research question (‘does a time limit alter the psychotherapeutic process?’), the literature points out that setting a time limit can certainly alter psychotherapeutic working mechanisms.

To summarize our findings, research that focuses on therapists’ experiences emphasizes that the intervention of setting a time limit influences their practice and forces them to adapt their therapeutic approach (Busch et al., 2001; Cohen et al., 2006; Wright et al., 2012). Practitioners indicate that a time limit sets pressure on the therapy process and creates a kind of expectancy effect (Wright et al., 2012), which can have both negative and positive consequences. On the one hand, the time limit was said to create a feeling of urgency and intensity in the therapy, which can speed up the process thus bringing up separation issues that would otherwise not come to the fore (this early on) in therapy (Busch et al., 2001). On the other hand, the intervention could harm the self-confidence of the therapist and create a situation in which practitioners cannot perform to the best of their abilities (Cohen et al., 2006; Wright et al., 2012). In this context, qualitative studies showed that therapists in TLP felt forced to provide therapies that were more superficial than their usual practice. They felt pressured to lay a focus in therapy, which induced a fear of not working in-depth enough. They also felt inclined to take on a more directive role and, therefore, worried to what extent the time limit undermined patients ability to take an active part in therapy (Cohen et al., 2006; Wright et al., 2012). Secondly, two studies showed clients to be dissatisfied with the implementation of a time limit in therapy (Dekker et al., 2005; Johnson & Gelso, 1980). Thirdly, regarding the speed of the process in TLP, several studies suggest that a shorter time limit enables quicker symptom improvement (Dekker et al., 2005; Shapiro et al., 2003). The interpretation of these findings however remains uncertain, as they might be influenced by answering tendencies such as the hello-goodbye effect. Moreover, this does not seem to hold true for changes at an interpersonal level (Dekker, et al., 2005). Finally, although several authors have suggested that a time limited feature in psychotherapy may cause lower dropout rates, the methodological problems and weaknesses in this research field have delayed the development of an answer to this question. Although a recent meta-analysis on dropout supported the hypothesis at first sight (Swift & Greenberg, 2012), the favorable dropout rates in TLP could be caused by other typical characteristics of the RCT setting, such as the preselection of patients with a single diagnosis. In the future, explanatory research is necessary to clear up this ambiguity.

Overall, the directive role on behalf of the therapist is a phenomenon that we saw returning throughout several topics in our results. More specifically, in the study of Shapiro et al. (2003) where a comparison was made between 8 and 16–sessions, therapists were found to focus earlier on change in the eight-session condition than in the 16-session condition. Additionally, regarding patient characteristics, we saw that the personality trait ‘dominance’ did not correspond well with TLP. The researchers suggested that the active stance of therapists in TLP was in discordance with this trait (Vinnars et al., 2007). These findings seem to indicate that is it hard to pinpoint a general influence of a time limit on psychotherapy, not taking into account the different effects it can have on various patient populations. In general, it is hard to imagine one type of therapy that fits all patients and the time limit could be a factor that interacts with these variations.

### Elaborations and suggestions for future research

Through the results of our review, we have discerned several factors that could play a role in the mechanisms of a time restricted therapy. However, more thorough empirical research is necessary to provide a clear overview of the different factors involved in TLP. Also, it seems essential for good clinical practice to discover which settings can be detrimental for TLP and which could prove beneficial. Grounded theory analysis on the mechanisms involved in TLP, most preferably applied in a variety of settings could help us obtain these answers. This kind of qualitative research could provide a first step towards a theory on TLP, after which more specific hypotheses could be looked into.

As we saw in our theme “Therapeutic approach is altered”, therapists found that their way of working was altered by the time limit. Future research could discern which variables play a role in creating a positive (productive focus) or a negative (detrimental tension) experience for the therapists. Moreover, the interaction with differences in therapeutic approach should be taken into account. We can hypothesize that cognitive behavioral therapists, who are used to taking on a more directive role in therapy, feel differently working under time constraints compared to psychodynamic or experiential therapists, who usually take up a more exploratory role.

The setting in which a time limit is imposed may also affect its influence. In our results, therapists’ opinions on TLP varied along different contexts. While in managed care, opinions were mostly negative, a different reaction was seen in a research context where the therapists also mentioned several positive effects of a time limit. Unfortunately, given the small amount of studies on the subject in our review, it is not possible to make decisive conclusions. We hypothesize that therapists may have different attitudes towards the intervention depending on how and by whom the time limit was introduced. For example, the time time limit could be conceded differently when it is accompanied by a clear rationale. Also, the amount of sessions should be taken into account, since there can be a difference in how the pressure of an eight-session time limit is experienced by therapists compared to a 20-session one.

The results of our review suggest that patiënts can feel uncomfortable when their therapy is restricted in time. An obvious explanation for this could be that they fear a shortage of time, and this causes anxiety. As we saw in our theme ‘client characteristics’, little is known about how different patient characteristics interact with a time limit in therapy. In general, the influence of patient characteristics has only been marginally explored in psychotherapy research (Bohart & Wade, 2013). At the same time, evidence suggests that clients make the single strongest contribution to outcome in psychotherapy (Bohart & Wade, 2013). Regarding clients’ attachment style, for example, it has been shown that clients’ global attachment styles can impact the outcome and process of psychotherapy (Bohart & Wade, 2013). Therefore, it is promising to explore how patient characteristics interact with a time limit in psychotherapy. For example, patients that have experienced many (recent) losses in their lives may handle the strict separation at the end of therapy differently compared to those who have not. Our review showed for example that the character trait “dominance” did not go together well with a manualized form of therapy. It would be interesting to study if other traits can affect this experience as well. As we saw in our introduction, research suggests that patient characteristics such as higher distress levels at intake, higher baseline distress and the presence of characterological symptoms could account for a slower rate of change in therapy (Baldwin et al., 2009). Transfering these ideas to the context TLP, this could mean that these features might not abide well with a (short) time limit.

Besides the diversity in therapist and patient characteristics, we can also explore the point where the two come together, namely the therapeutic alliance, which is commonly known to be an essential element of the therapeutic process (Martin, Garske, & Davis, 2000). A positive connection between the therapist and patient could induce a positive attitude of the patient towards the time limit, given that the therapist is in favor of the limit him or herself. On the other hand, certain authors question how much space is left to form a therapeutic alliance, when time is limited in advance (Cushman & Gilford, 2000).

As we discussed above, some therapists in the study of Wright, Simpson-Young and Lennings (2012) felt that the therapeutic alliance was threatened by the imposed time limits. Following this line of reasoning, it seems important to explore whether a time limit impoverishes the formation or depth of the therapeutic alliance.

The results of our review showed that a shorter time limit can cause an acceleration of change in symptom improvement for patients. These findings can be appealing for clinical practice and especially the managed care context, since ‘time is money’ and shorter ways to help clients are more than welcome. We should, however, consider whether these changes in sypmptom improvement stay effective on the long run. A recent study in Finland (Knekt, et al., 2016) made a comparison between long-term^8^ and time-limited psychodynamic psychotherapy^9^ (hereafter named LPP and TPP), for patients suffering from a depressive or anxiety disorder. The study had an exceptionally long follow-up period of 10 years and the results showed that after this time span, the prevalence of auxiliary treatment – both the use of therapy and psychotropic medication – was three times higher in the TPP group during the follow-up period. According to Westen and Bradley (2005), meta-analytic data on empirically supported treatments (ESTs) for a range of disorders suggests that the modal patient treated in brief treatment relapses or seeks additional treatment within 12 to 24 months. If time limits are thus installed to shorten therapy processes and cut the costs of therapy, we could well end up disappointed in the long run. We should therefore investigate the long-term effects of a therapy that is not intrinsically terminated by the patient and/or therapist. On the other hand, some practitioners and theorists promote the idea of ‘intermittent psychotherapy’, where several planned time-limited psychotherapies take place over an extended period of time (Drisko, 2005).

### Strengths and limitations

Despite systematic screening of the literature, we might have missed some studies that would be relevant in this context. We used two search engines (Web of Knowlegde, Pubmed) for our search. Augmenting the number of search engines could have yielded more results. Also, the fact that articles that show null findings are hard to publish in the current research climate implies that is impossible to report such studies. However, one of the strengths of this review lies within the fact that it is the first in its kind to focus specifically on the processes involved in TLP, rather than focus on outcome. Our results provide a unique combination of quantitative and qualitative findings to explore our research questions.

## Conclusion

It seems warranted to conclude that there is an scarcity of research on the working mechanisms of a time restricted therapy process. In general, the lack of research on the processes and mechanisms of psychotherapy is a missed opportunity. Although some themes (such as the therapeutic alliance, or therapist development (McLeod, 2013c)) have received more qualitative attention in the past few years, processes such as the time limited ending of psychotherapy remain obscure. The results of our study show that the process of TLP is a complex phenomenon with many different factors involved. Applying a time limit to psychotherapy may have advantages for the therapy process. The added deadline can add a sence of urgency and intensity that causes therapists and patients to make the most of time in therapy. This could cause an acceleration in symptom improvement and lower drop-out rates. However, our results also to emphasize possible disadvantages of a time limited therapy process. Therapists feel they have to provide a superficial therapy in wich they have to take on a more directive stance and a stricter focus. Also, clients seem hindered in taking an active role and could feel dissatisfied with having limited time in therapy.

## Footnotes

Unfortunately, the length of the time limits in those therapies were not discussed in the article.

The majority of the therapists reported to work eclectic. All participants reported using CBT in their therapy.

73% in the 8-session condition and 58% in the 16-session condition.

54% in the 8-session condition and 50% in the 16-session condition.

On the Beck Depression Inventory.

Through a semi-structured interview at weeks 4, 8, 12, 16 and 24.

At 8 weeks, 31% of the 8-session patients had already reached remission according to the HDRS, while remission had only been achieved in 18% of the 16-session patients. The same analysis was made for both the SCL-90 and QLDS (Dekker et al., 2005).

Open ended, +/– 3 years.

20 sessions.

## Appendix A. Focus, Type and Sample, and Summary of the Included Review Articles

**Table:**

Author(s)	Focus	Type and sample	Summary

Johnson & Gelso (1980)	T, P, PC	Meta-analysis Counselor & Client ratings	Counselors themselves felt less inclined towards TLP. Although TLP clients seemed to be satisfied with their counseling in general, multiple studies found them to be less satisfied with the length of treatment. Less well-adjusted, more chronic clients may do better in open-ended therapy than in TLP.
Phelps, Eisman, & Kohout (1998)	T	Quantitative Questionnaires Variety of therapeutic orientations	Majority of psychologists indicated managed care (including time limited facet) had a negative impact on their practices.
Burlingame & Behrman (1987)	T	Meta-analysis (36 studies) Clinician reports, Client reports, Questionnaires	TLP was perceived as more efficacious by therapists in cases of situational adjustment reactions, but they favored unlimited treatment over TLP for several other diagnoses.
Murphy, Debernardo, & Shoemaker (1998)	T	Quantitative Questionnaires N = 180	Majority of clinicians in a managed care setting indicated that putting limits on the number of sessions interfered with their treatment.
Cohen, Marecek, & Gillham (2006)	T	Qualitative Semi-structured interviews (N = 18) Variety of therapeutic orientations	2/3 of the therapists in a managed care setting indicated that the lack of control over the length of treatment was an obstacle to its successfulness. Time constraints made them alter their therapeutic approach.
Wright, Simpson-Young, & Lennings (2012)	T	Quantitative (n = 85) & Qualitative (n = 27) Questionnaires & Interviews Mostly CBT-therapists	Therapists in a managed care setting felt their therapeutic approach was impacted by time limits and they worked more superficial in a time limited treatment.
Busch et al. (2001)	T	Qualitative (N = 6) Psychoanalytic therapists	The time limit created pressure for therapists in a research setting, which had its up and downsides.
Dekker et al. (2005)	P, A	Quantitative Questionnaires (N = 103) Short Psychodynamic Supportive Therapy	43% of patients thought there were not enough therapy sessions. Results also suggested an acceleration of symptom reduction in the 8 session condition compared to the 16 session condition.
Shapiro (2003)	A	Experimental Multi-level measurement strategy (N = 117) CBT and Psychodynamic Psychotherapy	Results suggested an acceleration of symptom reduction in the 8 sessions condition compared to the 16 session condition.
Messer (2001)	A	Conceptual	Time limit is believed to accelerate the process of psychotherapy by increasing the sense of urgency, immediacy, and emotional presence of the patient, as well as the focus and activity of the therapist.
Eckert (1993)	A	Conceptual	Time limit is believed to accelerate the process of psychotherapy.
Rasmussen & Messer (1986)	A	Conceptual	Time limit is believed to accelerate the process of psychotherapy.
Hatcher, Huebner, & Zakin (1986)	A	Quantitative & Qualitative N = 47	The focus in TLP changes and evolves over the course of the treatment.
Migone (2014)	A	Conceptual	Pleads to understand why the patient needs to be pushed in order to work faster, and not to bypass this resistance with a parameter such as the time limit.
Swift & Greenberg (2012)	D	Meta-analysis (669 studies) N = 83.834	Higher drop-out rates can be found for open-ended than time-limited treatments.
Wierzbicki & Pekarik (1993)	D	Meta-analysis (125 studies)	High drop-out rates can be explained by the fact that clients might expect and desire a shorter treatment duration compared to what their therapists foresee for them.
Ogrodniczuk, Joyce, & Piper (2005)	D	Review (29 studies)	Lower dropout rates for short-term therapies could be a function of time itself: patients in short-term therapy have less occasion to terminate prematurely compared to patients in long-term therapy.
Pekarik (1985)	D	Conceptual	Explains lower drop-out rates in TLP by the fact that clients might expect and desire a shorter treatment duration compared to what their therapists foresee for them.
Straker (1968)	D	Empirical (N = 220)	Study that showed that TLP had lower drop-out rates than open ended treatment.
Reder & Tyson (1980)	D	Meta-analysis Conceptual	Suggests TLP has lower drop-out than open ended treatment.
Sledge, Moras, Hartley, & Levine (1990)	D	Empirical (N = 149) Variety of severity in diagnoses	Study that showed that TLP had lower drop-out rates than open ended treatment.
Deykin, Weissman, Tanner, & Prusoff (1975)	D	Quantitative (N = 36) Depressed female patients	The authors suggest that one of the variables that contributed to the unexpectedly high therapy attendance was the time limited nature of the therapy.
Mann (1991)	PC	Conceptual	Certain diagnostic categories (such as schizophrenia, bipolar disorder, and schizoid characters) a priori demand indefinite long-term involvement with the patient and therefore do not correspond well with a time limited format.
Ursano & Hales (1986)	PC	Conceptual	The authors indicate a number of exclusion criteria for TLP: severe depression, acute psychosis, borderline personality, and the inability to identify a central issue.
Vinnars et al. (2007)	PC	Quantitative Questionnaires (N = 156) Patients with a personality disorder	Suggests that the active stance of therapists in time-limited, manualized psychotherapy is not complementary with patients with highly dominant personality traits.
Thomas (2017)	PC	Qualitative Case study (n = 1) Time limited psychodynamic therapy	Describes the therapy process of working time limited with a patient diagnosed with schizophrenia.
Binder (1979)	PC	Qualitative Case study (n = 1) Time limited psychodynamic therapy	Describes the therapy process of working time limited with a patient diagnosed with narcissistic problems.
Thase et al. (1992)	PC	Quantitative (N = 48) Cognitive behavioral therapy	Variables that correlated to relapse after TLP included a history of depressive episodes, higher levels of depressive symptoms and dysfunctional attitudes and a slower response to therapy.

*Note*: T = Therapists alter their therapeutic approach; P = Patients’ experiences; PC = Patients’ characteristics; A = Acceleration of change; D = Drop-out.

## References

[1] Abbass, A., Kisely, S., Town, J., Leichsenring, F., Driessen, E., De Maat, S., Crowe, E., et al. (2014). Short-term psychodynamic psychotherapies for common mental disorders. Cochrane database of systematic reviews, 7 DOI: 10.1002/14651858.CD004687.pub4 PMC11129844 24984083

[2] Baldwin, A. S., Berkeljon, A., Atkins, D. C., Joseph, O. A., & Nielsen, L. S. (2009). Rates of change in naturalistic psychotherapy: Contrasting dose-effect and good-enough level models of change. Journal of Consulting and Clinical Psychology, 77(2), 203–211. DOI: 10.1037/a0015235 19309180

[3] Beck, A. T., Ward, C. H., Mendelson, M., Mock, J., & Erbaugh, J. (1961). An inventory for measuring depression. Archives of General Psychiatry, 4, 561–571. DOI: 10.1001/archpsyc.1961.01710120031004 13688369

[4] Binder, J. L. (1979). Treatment of narcissistic problems in time-limited psychotherapy. Psychiatric Quarterly, 51(4), 257–70. DOI: 10.1007/BF01082829 523572

[5] Blais, M. A., & Hilsenroth, M. J. (2007). Methodcentric reasoning and the empirically supported treatment debates In Hofmann, S. G., & Weinberger, J. (Eds.), The art and science of psychotherapy (pp. 31–48). New York: Routledge.

[6] Bohart, A. C., & Wade, A. G. (2013). The client in psychotherapy In Lambert, M. J. (Ed.), Handbook of psychotherapy and behavior change (pp. 219–257). USA: Wiley.

[7] Braun, V., & Clarke, V. (2006). Using thematic analysis in psychology. Qualitative Research in Psychology, 3(2), 77–101. DOI: 10.1191/1478088706qp063oa

[8] Burlingame, G., & Behrman, J. (1987). Clinician attitudes toward time-limited an time-unlimited therapy. Professional psychology – Research and practice, 18(1), 61–65. DOI: 10.1037//0735-7028.18.1.61

[9] Busch, F., Milrod, B., Rudden, M., Shapiro, T., Roiphe, J., Singer, M., & Aronson, A. (2001). How treating psychoanalysts respond to psychotherapy research constraints. Journal of the American psychoanalytic association, 49(3), 961–984. DOI: 10.1177/00030651010490030601 11678245

[10] Cohen, J., Marecek, J., & Gillham, J. (2006). Is three a crowd? Clients, clinicians, and managed care. Americal journal of orthopsychiatry, 76(2), 251–259. DOI: 10.1037/0002-9432.76.2.251 16719644

[11] Dekker, J., Molenaar, P., Kool, S., Van Aalst, G., Peen, J., & de Jonghe, F. (2005). Dose-effect relations in time-limited combined psycho-pharmacological treatment for depression. Psychological Medicine, 35(1), 47–58. DOI: 10.1017/S0033291704002685 15842028

[12] Deykin, E., Weissman, M., Tanner, J., & Prusoff, B. (1975). Participation in therapy. A study of attendance patterns in depressed outpatients. The Journal of Nervous and Mental Disease, 160(1), 42–48. DOI: 10.1097/00005053-197501000-00007 1113093

[13] Drisko, J. (2005). Intermittent psychotherapy. Smith college studies in social work, 75(2), 7–25. DOI: 10.1300/J497v75n02_02

[14] Eckert, P. (1993). Acceleration of change – Catalysts in brief therapy. Clinical psychology review, 13(3), 241–253. DOI: 10.1016/0272-7358(93)90022-E

[15] Feaster, D., Newman, F., & Rice, C. (2003). Longitudinal analysis when the experimenter does not determine when treatment ends: What is dose-response? Clinical psychology & psychotherapy, 10(6), 352–360. DOI: 10.1002/cpp.382 16724159 PMC1467574

[16] Freud, S. (1918). From the history of an infantile neurosis In Trachey, J. (Ed.), The Standard Edition of the Complete Psychological Works of Sigmund Freud (vol. 17, pp. 1–122). London: Hogarth Press.

[17] Groves, J. (1996). Introduction: Four “Essences” of Short-Term Therapy: Brevity, Focus, Activity, Selectivity In Groves, J. E. (Ed.), Essential Papers on Short-Term Dynamic Therapy (pp. 1–26). New York: University Press.

[18] Hansen, B. N., Lambert, J. M., & Forman, M. E. (2002). The psychotherapy dose-response effect and its implications for treatment delivery services. Cinical psychology – Science and practice, 9(3), 329–343. DOI: 10.1093/clipsy/9.3.329

[19] Hatcher, S., Huebner, D., & Zakin, D. (1986). Following the trail of focus in time-limited psychotherapy. Psychotherapy, 23(4), 513–520. DOI: 10.1037/h0085650

[20] Hill, C., CHui, H., & Baumann, E. (2013). Revisiting and reenvisioning the outcome problem in psychotherapy: An argument to include Individualized and qualitative measurement. Psychotherapy, 50(2), 166–166. DOI: 10.1037/a0033226 23505982

[21] Horowitz, L. M., Rosenberg, S. E., Baer, B. A., Ureno, G., & Villasenor, V. S. (1988). Inventory of interpersonal problems: Psychometric properties and clinical applications. Journal of Consulting and Clinical Psychology, 56, 885–892. DOI: 10.1037/0022-006X.56.6.885 3204198

[22] Howard, K. I., Kopta, S. M., Krause, M. S., & Orlinsky, E. D. (1986). The dose-effect relationship in psychotherapy. American Psychologist, 41(2), 159–164. DOI: 10.1037//0003-066X.41.2.159 3516036

[23] Hoyt, M. F. (2005). Brief Psychotherapies In Messer, S. B., & Gurman, A. S. (Eds.), Essential Psychotherapies (pp. 350–399). New York: The Guilford Press.

[24] Johnson, D., & Gelso, C. (1980). The effectiveness of time limits in counseling and psychotherapy – A critical review. Counseling Psychologist, 9(1), 70–83. DOI: 10.1177/001100008000900115

[25] Knekt, P., Virtala, E., Harkanen, T., Vaarama, M., Lehtonen, J., & Lindfors, O. (2016). The outcome of short- and long-term psychotherapy 10 years after start of treatment. Psychological medicine, 46(6), 1175–1188. DOI: 10.1017/S0033291715002718 26755201

[26] Malan, D. (1976). The frontier of brief psychotherapy. New York: Plenum Press DOI: 10.1007/978-1-4684-2220-7

[27] Mann, J. (1973). Time-Limited psychotherapy. London: Harvard University Press.

[28] Mann, J. (1991). Time limited psychotherapy – Handbook of short-term dynamic psychotherapy. USA: Basic Books Retrieved from https://www.israpsych.org/books/wp-content/uploads/2016/01/time_limited_psychotherapy.pdf

[29] Martin, D. J., Garske, J. P., & Davis, K. M. (2000). Relation of the therapeutic alliance with outcome and other variables: A meta-analytic review. Journal of consulting and clinical psychology, 68(3), 438–450. DOI: 10.1037/0022-006X.68.3.438 10883561

[30] McLeod, J. (2013b). Increasing the rigor of case study evidence in therapy research. Pragmatic case studies in psychotherapy, 9(4), 382–402. DOI: 10.14713/pcsp.v9i4.1832

[31] McLeod, J. (2013c). Qualitative research – Methods and Contributions In Lambert, M. J. (Ed.), Handbook of psychotherapy and behavior change (pp. 49–84). USA: Wiley.

[32] Messer, S. (2001). What allows therapy to be brief? Introduction to the special series. Clinical psychology – science and practice, 8(1), 1–4. DOI: 10.1093/clipsy/8.1.1

[33] Migone, P. (2014). What does “brief” mean? A theoretical critique of the concept of brief therapy from a psychoanalytic viewpoint. Journal of the American psychoanalytic association, 62(4), 631–656. DOI: 10.1177/0003065114544319 25049047

[34] Murphy, M., Debernardo, C., & Shoemaker, W. (1998). Impact of managed care on independent practice and professional ethics: A survey of independent practitioners. Professional psychology – Research and practice, 29(1), 43–51. DOI: 10.1037/0735-7028.29.1.43 11660484

[35] Ogrodniczuk, J., Joyce, A., & Piper, W. (2005). Strategies for reducing patient-initiated premature termination of psychotherapy. Harvard review of psychiatry, 13(2), 57–70. DOI: 10.1080/10673220590956429 16020021

[36] Pekarik, G. (1985). Coping with dropouts. Professional psychology – Research and practice, 16(1), 114–123. DOI: 10.1037/0735-7028.16.1.114

[37] Phelps, R., Eisman, E., & Kohout, J. (1998). Psychological practice and managed care: Results of the CAPP practitioner survey. Professional psychology – Research and practice, 29(1), 31–36. DOI: 10.1037//0735-7028.29.1.31

[38] Rasmussen, A., & Messer, S. (1986). A comparison and critique of Mann time-limited psychotherapy and Davanloo short-term dynamic psychotherapy. Bulletin of the Menninger Clinic, 50(2), 163–184. 3955280

[39] Reder, P., & Tyson, R. L. (1980). Patient dropout from individual psychotherapy. Bulletin of the Menninger Clinic, 44(3), 229–252. 7388191

[40] Roos, J., & Werbart, A. (2013). Therapist and relationship factors influencing dropout from individual psychotherapy: A literature review. Psychotherapy research, 23(4), 394–418. DOI: 10.1080/10503307.2013.775528 23461273

[41] Safran, J. (2002a). Brief relational psychonalytic treatment. Psychoanalytic Dialogues, 12(2), 171–195. DOI: 10.1080/10481881209348661

[42] Safran, J. (2002b). Reply to Commentaries. Psychoanalytic Dialogues, 12(2), 235–258. DOI: 10.1080/10481881209348665

[43] Shapiro, D., Barkham, M., Stiles, W., Hardy, G., Rees, A., Reynolds, S., & Startup, M. (2003). Time is of the essence: A selective review of the fall and rise of brief therapy research. Psychology and psychotherapy – Theory research and practice, 76, 211–235. DOI: 10.1348/147608303322362460 14577890

[44] Sifneos, P. (1979). Short term dynamic psychotherapy: Evaluations and technique. New York: Plenum Press DOI: 10.1007/978-1-4684-3530-6

[45] Sledge, W., Moras, K., Hartley, D., & Levine, M. (1990). Effect of time-limited psychotherapy on patient dropout rates. American journal of psychiatry, 147(10), 1341–1347. DOI: 10.1176/ajp.147.10.1341 2400003

[46] Straker, M. (1968). Brief Psychotherapy in an Outpatient Clinic: Evolution and Evaluation. American Journal of Psychiatry, 124(9), 105–112. DOI: 10.1176/ajp.124.9.1219 4866652

[47] Stulz, N., Kopta, M. S., Lutz, W., Minami, T., & Saunders, M. S. (2013). Dose-Effect relationship in Routine Outpatient Psychotherapy: Does treatment Duration Matter? Journal of Counseling Psychology, 593–600. DOI: 10.1037/a0033589 23815633

[48] Swift, J., & Greenberg, R. (2012). Premature discontinuation in adult psychotherapy: A meta-analysis. Journal of consulting and clinical psychology, 80(4), 547–559. DOI: 10.1037/a0028226 22506792

[49] Thase, M. E., Simons, A. D., McGeary, J., Cahalane, J. F., Hughes, C., Harden, T., & Friedman, E. (1992). Relapse after cognitive behavior therapy of depression: potential implications for longer courses of treatment. American Journal of Psychiatry, 149(8), 1046–52. DOI: 10.1176/ajp.149.8.1046 1636804

[50] Thomas, Z. (2017). Breaking Through to the Other Side: A Resident Explores the Benefits of Time-Limited Psychodynamic Therapy for Patients with Schizophrenia. Psychodynamic Psychiatry, 45(1), 59–77. DOI: 10.1521/pdps.2017.45.1.59 28248562

[51] Ursano, R. J., & Hales, R. E. (1986). A review of brief individual psychotherapies. American Journal of Psychiatry. 143(12), 1507–1517. DOI: 10.1176/ajp.143.12.1507 3538911

[52] Vinnars, B., Barber, J., Noren, K., Thormahlen, B., Gallop, R., Lindgren, A., & Weinryb, R. M. (2007). Who can benefit from time-limited dynamic psychotherapy? A study of psychiatric outpatients with personality disorders. Clinical psychology & psychotherapy, 14(3), 198–210. DOI: 10.1002/cpp.530

[53] Westen, D., & Bradley, R. (2005). Empirically supported complexity – Rethinking evidence-based practice in psychotherapy. Current directions in psychological science, 14(5), 266–271. DOI: 10.1111/j.0963-7214.2005.00378.x

[54] Wierzbicki, M., & Pekarik, G. (1993). A meta-analysis of psychotherapy dropout. Professional psychology – Research and practice, 24(2), 190–195. DOI: 10.1037/0735-7028.24.2.190

[55] Wright, T., Simpson-Young, V., & Lennings, C. (2012). Therapeutic process in the context of third party determined time limits. Clinical Psychologist, 16(2), 82–92. DOI: 10.1111/j.1742-9552.2012.00043.x

